# Human papillomavirus-related oropharyngeal squamous cell carcinoma exhibits enhanced radiosensitivity despite limited activation of cytosolic DNA sensing pathways and innate immune responses

**DOI:** 10.2478/raon-2025-0057

**Published:** 2025-12-16

**Authors:** Kristina Levpuscek, Tanja Jesenko, Tilen Komel, Simona Kranjc Brezar, Gregor Sersa, Maja Cemazar, Primoz Strojan

**Affiliations:** 1 Department of Experimental Oncology, Institute of Oncology Ljubljana, Ljubljana, Slovenia; 2 Faculty of Medicine, University of Ljubljana, Ljubljana, Slovenia; 3 Faculty of Mathematics, Natural Sciences and Information Technologies, University of Primorska, Koper, Slovenia; 4 Faculty of Health Sciences, University of Ljubljana, Ljubljana, Slovenia; 5 Faculty of Health Sciences, University of Primorska, Koper, Slovenia; 6 Department of Radiation Oncology, Institute of Oncology, Ljubljana, Slovenia

**Keywords:** cytosolic DNA sensing pathways, innate immune response, human papillomavirus type 16, pharyngeal squamous cell carcinoma, radiation responseIntroduction

## Abstract

**Background:**

Pharyngeal squamous cell carcinoma (PSCC) is a significant health concern, with human papillomavirus 16 (HPV16) playing a key role in the etiology of oropharyngeal squamous cell carcinoma (OPSCC). HPV16-related OPSCC exhibits enhanced radiosensitivity compared to HPV16-unrelated PSCC, yet the underlying mechanisms remain poorly understood. As HPV16 oncoproteins E6 and E7 are known to interfere with innate immune signaling, we investigated how modulation of cytosolic DNA sensing pathways and innate immune responses changes after irradiation (IR) and whether this contributes to enhanced radiosensitivity in HPV16-related OPSCC.

**Materials and methods:**

Using HPV16-related and -unrelated PSCC models, we examined baseline expression levels of DNA sensors and cytokines and assessed the effects of IR on double-stranded DNA (dsDNA) accumulation, activation of cytosolic DNA sensors, cytokines, and immune cell infiltration both *in vitro* and *in vivo*. Analyses were performed using real-time quantitative polymerase chain reaction (RT-qPCR) and immunofluorescent staining.

**Results:**

HPV16-related OPSCC exhibited a distinct baseline expression profile of DNA sensors and cytokines, consistent with suppression of the stimulator of interferon genes (STING) pathway. While IR-induced activation of DNA sensors was dose- and time-dependent across models, HPV16-related OPSCC showed selective activation of cyclic GMP-AMP synthase (cGAS) and STING without significant cytokine upregulation or immune activation. In contrast, HPV16-related and unrelated PSCCs displayed activation of multiple DNA sensors, increased cytokine expression, and enhanced immune cell infiltration following IR.

**Conclusions:**

The key finding was that the involvement of cytosolic DNA sensing pathways and innate immune system do not increase radiosensitivity of HPV16-related OPSCC. In PSCC models, DNA sensor and cytokine expression varied depending on IR dose and fractionation.

## Introduction

Head and neck squamous cell carcinomas (HNSCCs) arise from the mucosal epithelium of the upper aerodigestive tract and represent the seventh most common cancer worldwide, with an estimated 800,000 new cases and 400,000 deaths in 2022.^1^ HNSCCs are typically associated with excessive alcohol and tobacco use, while oropharyngeal squamous cell carcinoma (OPSCC) is increasingly linked to infection with human papillomavirus type 16 (HPV16).2,3 HPV16 belongs to the Papillomaviridae family and is classified as a high-risk oncogenic type.^4^ Epidemiological studies have shown a decline in the incidence of HPV16-unrelated HNSCC, whereas HPV16-related OPSCC is on the rise.^5,6^ Standard treatments for HNSCCs include surgery, radiotherapy (RT), and chemotherapy. The survival rate has seen modest improvements over the last three decades. Previous studies have shown higher response rates to RT and chemotherapy and consequently improved survival for patients with HPV16-related OPSCC compared to those with HPV16-unrelated tumors.^7–9^ However, the molecular and immunological mechanisms underlying this enhanced radiosensitivity remain poorly understood.

The innate immune response to ionizing radiation (IR) is emerging as a critical factor influencing tumor radiosensitivity. IR-induced DNA damage can lead to cytosolic accumulation of double-stranded DNA (dsDNA), which activates the cytosolic DNA sensing pathways such as cyclic GMP-AMP synthase (cGAS). cGAS detects dsDNA and produces cyclic GMP-AMP (cGAMP), a second messenger that binds to and activates the stimulator of interferon genes (STING). This leads to activation of downstream signaling pathways and ultimately the production of type I interferons (IFN-I) and other pro-inflammatory cytokines that drive the innate immune response against damaged or malignant cells. These pathways are also part of the fundamental mechanism of host defense.^10–13^

There is increasing evidence that HPV16 oncoproteins E6 and E7 disrupt host innate immune signaling to facilitate immune evasion and promote carcinogenesis. Studies have shown that these oncoproteins suppress several DNA sensors, including retinoic acid-inducible gene I (RIG-I) and Toll-like receptors (TLRs), and to directly inhibit the cGAS-STING axis in HPV16-related OPSCC.^14,15^ This suppression impairs the production of IFN-I, which may enable infected cells to escape immune surveillance. The host immune response is a critical component of antitumor immunity. Therefore, immune dysregulation by HPV16 may significantly influence the efficacy of RT.^16^

In this study, we investigated how the HPV16 oncoproteins E6 and E7 modulate the activation of cytosolic DNA sensing pathways and innate immune response following IR in pharyngeal squamous cell carcinomas (PSCCs) models, and whether this modulation contributes to the enhanced radiosensitivity observed in HPV16-related OPSCC. Specifically, we assessed baseline expression and IR-induced activation of cytosolic DNA sensors, cytokine production, and innate immune cell infiltration in both tumor cells and the tumor microenvironment (TME), to better understand the immune landscape underlying the differential radiosensitivity of HPV16-related OPSCC.

## Materials and methods

### Cell lines

The *in vitro* experiments were performed with four human PSCC cell lines: HPV16-related OPSCC UPCI:SCC090 (RRID:CVCL_1899; ATCC^®^ CRL-3239™, Manassas, VA, USA), HPV16-unrelated OPSCC UM-SCC-6 (RRID:CVCL_7773; Merck-Millipore, Burlington, MA, USA), HPV16-related hypopharyngeal squamous cell carcinoma (HPSCC) 2A3 (RRID:CVCL_0D71; ATCC^®^ CRL-3212™, ATCC), and HPV16-unrelated HPSCC FaDu (RRID:CVCL_1218; ATCC^®^ HTB-43™, ATCC). All cell lines were cultured at 37°C in a humidified atmosphere containing 5% CO_2_ and were used within ten passages. UPCI:SCC090 and UM-SCC-6 cells were maintained in Advanced Dulbecco’s Modified Eagle’s Medium (ADMEM, Gibco, Thermo Fisher Scientific, Waltham, MA, USA). FaDu cells were cultured in Advanced Minimum Essential Medium (AMEM, Gibco), and 2A3 cells in ADMEM supplemented with 0.2 mg/ml G418 disulfate salt solution (Sigma-Aldrich LLC, St. Louis, MO, USA). All media were supplemented with 5% fetal bovine serum (FBS; Thermo Fisher Scientific), 1% GlutaMAX (Thermo Fisher Scientific), and 1% PenicillinStreptomycin (Merck). Cells were routinely tested with MycoAlert™ PLUS Mycoplasma Detection Kit (Lonza, Basel, Switzerland). All experiments were performed with mycoplasma-free cells.

### Experimental animals and tumor induction

*In vivo* experiments were performed on 8-week-old female Athymic Nude mice (Charles River, Lecco, Italy), housed in sterile cages under a 12-hour light/dark cycle with controlled temperature and humidity, and provided water and food ad libitum. All procedures were approved by the Ministry of Agriculture, Forestry and Food of the Republic of Slovenia (permission No. U34401-33/2019/9 and U34401-35/2020/8) in accordance with EU directive 2010/63/EU. Subcutaneous tumors were established by injecting 100 μL of 0.9% NaCl containing 5×106 viable UPCI:SCC090 cells, 10×10^6^ UM-SCC-6 cells, or 2×106 FaDu or 2A3 cells into the dorsal flank of mice. UM-SCC-6 tumor induction was unsuccessful despite attempts with various cell concentrations (1×10^6^, 3×10^6^ or 10×10^6^) and co-injection with basement membrane matrix (Corning^®^ Matrigel^®^ Matrix, Corning, New York, USA). Once tumors reached a volume of approximately 45-50 mm^3^, mice were distributed into different treatment groups.

### Irradiation

IR was performed using a Gulmay CP225 X-Ray Generator (Gulmay Medical Ltd., Byfleet, UK) at 200 kV and 9.2 mA, with a dose rate of 1.96 Gy/min. For *in vitro* experiments, cells were IR with 4 Gy, 8 Gy, or a fractionated dose of 3×8 Gy. For *in vivo* studies, tumor-bearing mice were immobilized in custom-designed lead holders with apertures allowing localized tumor IR. Mice received either a single dose of 8 Gy or a fractionated regimen of 3×8 Gy.

### Cell viability assay

Post-IR cell viability was evaluated using a resazurin-based assay (PrestoBlue™, Thermo Fisher Scientific). Cells were seeded in 96-well plates (VWR, Radnor, Pennsylvania, US) and allowed to adhere overnight prior to IR. Viability was assessed after four population doublings, accounting for doubling times of 24 h (FaDu, 2A3, UM-SCC-6) and four days (UPCI:SCC090). 10 μl PrestoBlue reagent was added per well, followed by a 1-hour incubation at 37°C in a humidified 5% CO_2_ atmosphere. Fluorescence intensity was measured using a microplate reader (GEN-ios, Tecan, Männedorf, Switzerland).

### Tumor growth measurement

Mice were distributed in experimental groups of 6 animals: control group, group irradiated with 8 Gy, or 3×8 Gy. Tumors were measured three times per week using a Vernier caliper, and volumes were calculated as V = a × b × c × π/6 (a, b, c representing tumor diameters). Mice were humanely euthanized when their tumor volumes reached 500 mm^3^, a threshold established as a humane endpoint for the Kaplan-Meier survival analysis. Complete response was defined as the absence of detectable tumors for 100 days

### Tumor collection

Mice were euthanized 72 hours post-IR alongside their respective control groups. Tumors were excised. One-half of the tumor was fixed in 4% paraformaldehyde for 12 hours, then immersed in 30% sucrose for 24 hours, embedded in Optimal Cutting Temperature (OCT; VWR) compound, and flash-frozen in liquid nitrogen for immunofluorescence analysis. The other half was flash-frozen, pulverized, and stored at –80°C for subsequent RNA extraction.

### Real-time quantitative polymerase chain reaction (RT-qPCR)

For the *in vitro* study, cells were seeded in T25 flasks (Corning), allowed to adhere, and then irradiated with 4, 8, or 3×8 Gy, except for the control group, which was also used to determine baseline expression of DNA sensing pathway genes. RNA was extracted at 48- or 72-hours post-IR using the peqGOLD Total RNA Kit (VWR, West Chester, PA, USA), following the manufacturer’s instructions. For tumor samples, TRIzol Reagent (Thermo Fischer Scientific) was used for homogenization and extraction, followed by isolation of RNA. SuperScript VILO cDNA Synthesis Kit (Invitrogen, Thermo Fisher Scientific) was used for reverse transcription. RT-qPCR was performed on a QuantStudio 3 Real-Time PCR System (Thermo Fisher Scientific). The samples were prepared using PowerUp SYBR Green Master Mix (Thermo Fisher Scientific) and predesigned primers specific for human or mouse DNA sensors and cytokines (IDT, IA, USA). Mouse-specific primers enabled discrimination of TME components. Relative expression was calculated using the ΔCq method: Cq (gene of interest)-Cq (mean of housekeeping genes). Fold changes were determined using the 2-ΔΔCt method.^17^ Non-determined (N.D.) values were defined as Cq > 40. Detailed protocols and primer sequences are listed in Supplementary materials and Supplementary Table S1.

### Accumulation of cytosolic dsDNA

Cells were seeded overnight in 12-well chamber slides (Ibidi, Gräfelfing, Germany) and IR with 4, 8, or 3×8 Gy. Controls remained unirradiated. After 48 or 72 hours, cells were stained with antibodies. Immunofluorescence microscopy was performed using an LSM 800 confocal microscope (Carl Zeiss, Oberkochen, Germany), and images were analyzed using Imaris software (Bitplane, Zurich, Switzerland). Antibody details and protocols are provided in Supplementary materials and Supplementary Table S2.

### Tumor immunofluorescence staining

Tumor sections were prepared using a Leica CM1850 cryostat (Leica Biosystems, Wetzlar, Germany), mounted on Superfrost Plus glass slides (ThermoFisher Scientific), and stained with antibodies (Supplementary Table S3). Imaging was performed using an LSM 800 confocal microscope (Carl Zeiss), and image analysis was carried out using Imaris (Bitplane) and CellProfiler software (Broad Institute, Cambridge, MA, USA). Antibodies and staining protocols are listed in Supplementary materials and Supplementary Table S3.

### Statistical analysis

Statistical analyses and data visualization were performed using GraphPad Prism version 9 (GraphPad Software, San Diego, CA, USA). All *in vitro* experiments were repeated three times unless otherwise stated. *In vivo* experiments were carried out once following the principles of the 3Rs. Data normality was assessed by the Shapiro-Wilk test. A two-tailed Student’s t-test and One-way analysis of variance (ANOVA) were used to evaluate the statistical significance between different groups, followed by post hoc test or non-parametric data, the Kruskal-Wallis test with post hoc analysis was used. Kaplan-Meier survival curves were analyzed using a Log-rank test. Statistical significance was defined as p < 0.05.

## Results

### HPV16-related OPSCC exhibits enhanced radiosensitivity

**FIGURE 1.**
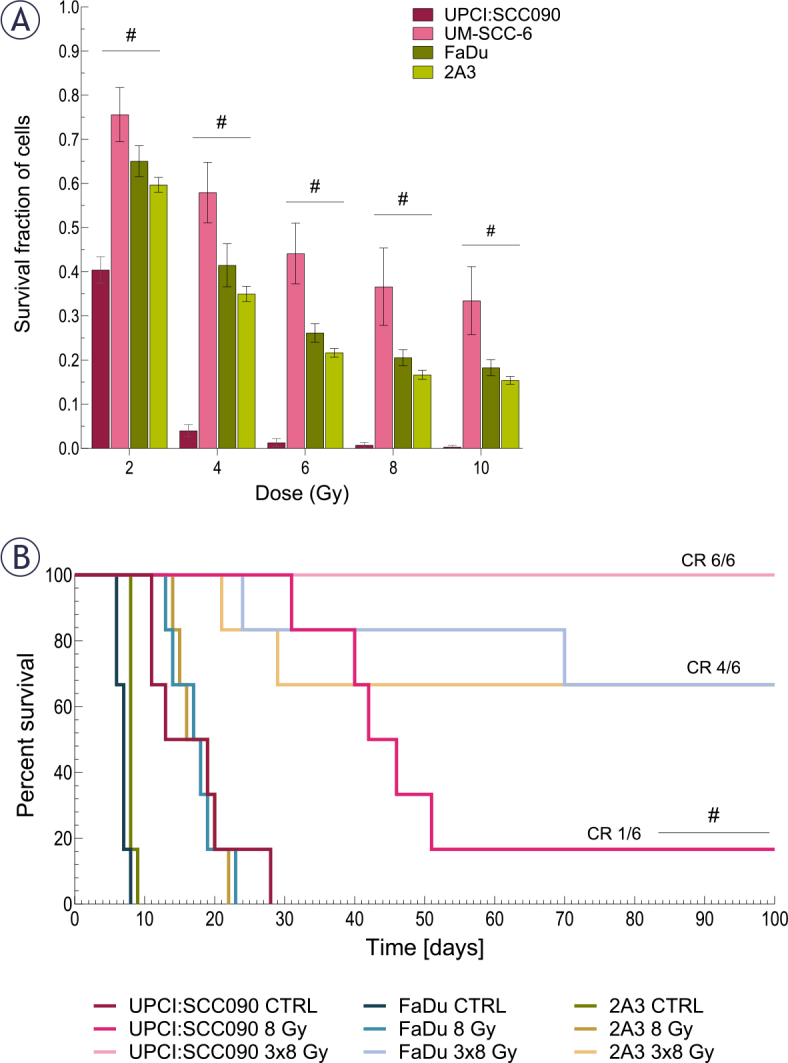
The effect of irradiation (IR) on cell survival and tumor growth. **(A)** Survival of cells after *in vitro* IR with 2, 4, 6, 8, and 10 Gy (n = 3). **(B)** Kaplan-Meier survival curve for mice bearing pharyngeal squamous cell carcinoma (PSCC) tumors treated with either a single dose of 8 Gy or 3×8 Gy, complete response (CR) (n = 6). Data are presented as mean ± standard error of the mean (SEM); # = indicates p < 0.05 for comparisons between UPCI:SCC090 and other cell lines or tumor models; * = indicates p < 0.05 for comparisons between IR doses within the same cell line or tumor model.

We evaluated the effect of IR on the survival of various cell lines and mice bearing PSCC tumors. Our *in vitro* findings showed that the HPV16-related OPSCC cell line UPCI:SCC090 had a better response to IR than the HPV16-unrelated OPSCC cell line UM-SCC-6 and both HPSCC cell lines. No notable differences in radiosensitivity were detected among the other PSCC lines (Figure 1A). We also examined the effect of IR on survival in mice bearing different PSCC tumors. Mice with UPCI:SCC090 tumors showed significantly better survival after a single dose of 8 Gy compared to the other two models. Notably, this group exhibited one complete response, a phenomenon not observed in the other models. In contrast, no differences in survival were observed following irradiation with 3×8 Gy, as all groups showed high rate of tumor cures. All UPCI:SCC090-bearing mice were cured, while in the FaDu and 2A3 models, four out of six mice achieved complete response (Figure 1B).

### Distinct baseline expression patterns of cytosolic DNA sensors and cytokines in HPV16-related OPSCC

**FIGURE 2.**
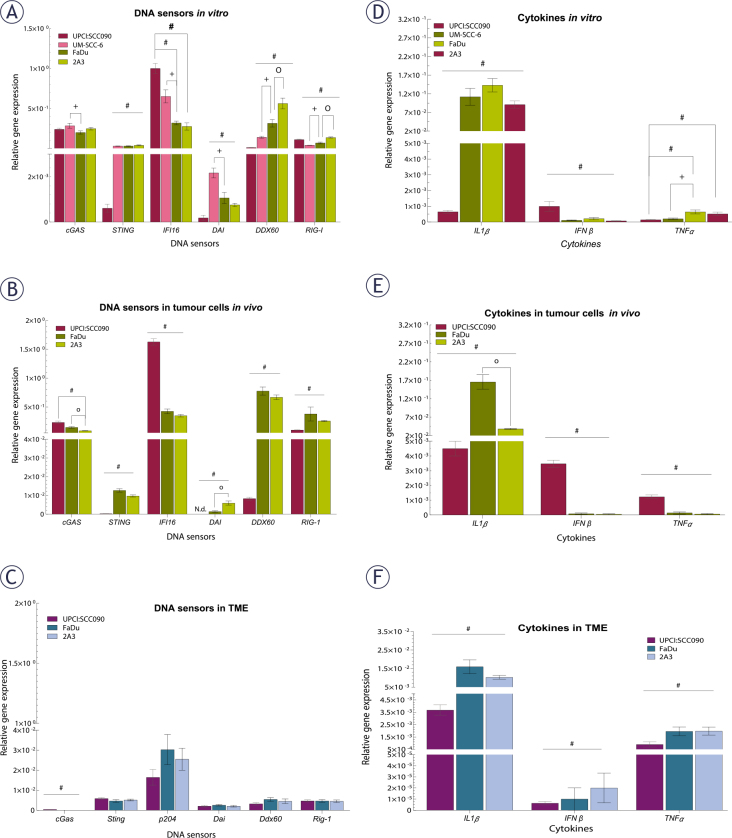
Baseline expression of cytosolic DNA sensors and cytokines in tumor cells and tumor microenvironment (TME) of pharyngeal squamous cell carcinomas (PSCCs). **(A)** Relative gene expression of cytosolic DNA sensors in cells *in vitro*, normalized to housekeeping genes (GUSB and B2M) (n = 3). **(B)** Relative gene expression of cytosolic DNA sensors in tumor cells *in vivo*, normalized to housekeeping genes (GUSB and B2M) (n = 5). **(C)** Relative gene expression of cytosolic DNA sensors in TME, normalized to housekeeping genes (BA and GADP) (n = 5). **(D)** Relative gene expression of cytokines in cells *in vitro*, normalized to housekeeping genes (GUSB and B2M) (n = 3). **(E)** Relative gene expression of cytokines in tumor cells *in vivo*, normalized to housekeeping genes (GUSB and B2M) (n = 5). **(F)** Relative gene expression of cytokines in TME, normalized to housekeeping genes (BA and GADP) (n = 5). Data is represented as mean ± standard error of the mean (SEM). # = indicates p < 0.05 for comparisons between UPCI:SCC090 and other cell lines or tumor models; * = indicates p < 0.05 for comparisons between irradiation (IR) doses within the same cell line or tumor model; o = indicates p < 0.05 for comparisons between FaDu and 2A3 models; + = indicates p < 0.05 for comparisons between UM-SCC-6 and FaDu

Our next step was to investigate the baseline expression of cytosolic DNA sensing pathways in PSCC tumor cells, both *in vitro* and *in vivo*, as well as in the TME. Baseline expression of cytosolic DNA sensors STING, DAI and DDX60 in UPCI:SCC090 cells was significantly lower compared to other PSCC cell lines, both *in vitro* and in vivo (Figures 2A–B). In contrast, IFI16 expression was elevated in UPCI:SCC090. When comparing the HPSCC cell lines FaDu and 2A3 *in vitro*, differences were observed in the expression levels of DDX60 and RIG-I (Figure 2A), while *in vivo* models differed in cGAS and DAI expression in tumor cells (Figure 2B). For the HPV16-unrelated cell lines UM-SCC-6 and FaDu, significant differences were detected in the expression of cGAS, IFI16, DAI, DDX60, and RIG-I, except for STING (Figure 2A). In the TME, baseline expression of cytosolic DNA sensors did not differ significantly between tumor models regardless of HPV16 status, except for cGas (Figure 2C). Furthermore, the expression levels of cytosolic DNA sensors in the TME were generally lower than those in the tumor cells (Figure 2A–C). Regarding cytokines, IL1β expression level was significantly lower in UPCI:SCC090 both *in vitro* and *in vivo* when compared to other PSCC models (Figure 2D–E). In contrast, IFNβ expression in tumor cells was significantly higher in UPCI:SCC090. The expression level of tumor necrosis factor (TNF)α in UPCI:SCC090 differed in *in vitro* compared to *in vivo* experiments (Figure 2D–E). In the TME of UPCI:SCC090, all cytokine levels were significantly lower compared to HPSCC tumor models (Figure 2F). No significant cytokine expression differences were observed between the two HPSCC models in the TME (Figure 2F).

### Limited activation of cytosolic DNA sensors following irradiation in HPV16-related OPSCC

**FIGURE 3.**
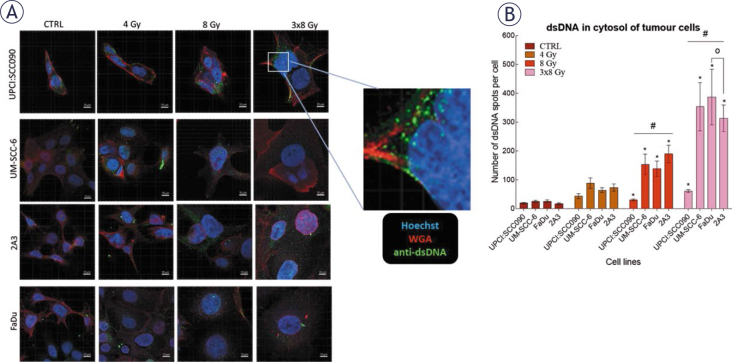
Irradiation (IR)-induced accumulation of dsDNA in the cytosol of pharyngeal squamous cell carcinoma (PSCC) cells. **(A)** Accumulation of dsDNA in the cytosol of cells 72 hours after IR. Green: dsDNA (anti-dsDNA), red: plasma membrane (WGA), blue: nucleus (Hoechst 33342), Scale bar = 10 μm. **(B)** Number of dsDNA spots per cell in cytosol 72 hours after IR with 4, 8, or 3x8 Gy (n = 8). Data is represented as mean ± standard error of the mean (SEM). # = indicates p < 0.05 for comparisons between UPCI:SCC090 and other cell lines or tumor models; * = indicates p < 0.05 for comparisons between IR doses within the same cell line or tumor model; o = indicates p < 0.05 for comparisons between FaDu and 2A3 models; + = indicates p < 0.05 for comparisons between UM-SCC-6 and FaDu

**FIGURE 4.**
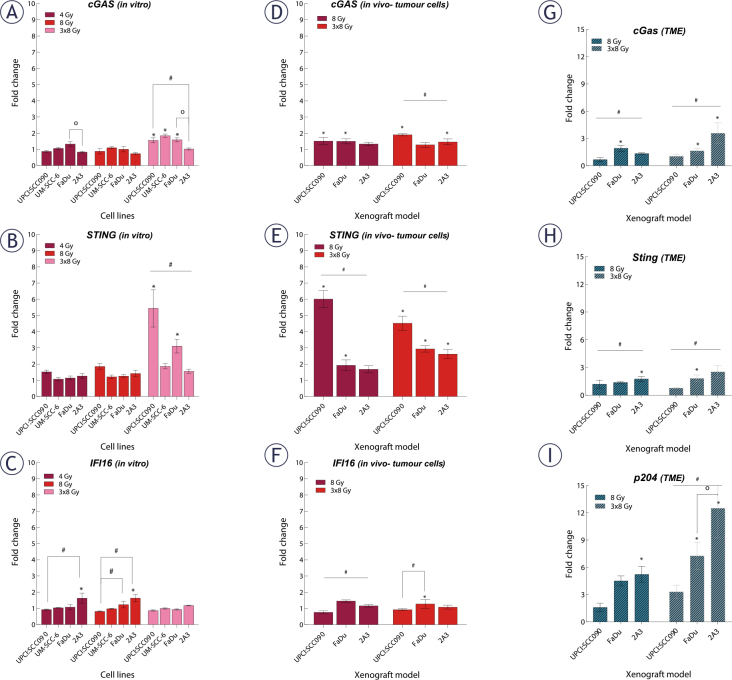
Effect of irradiation (IR) on cytosolic DNA sensors gene expression in tumor cells and and tumor microenvironment (TME) of pharyngeal squamous cell carcinoma (PSCC). **(A–C)** Fold change in expression of cyclic GMP-AMP synthase (cGAS) **(A)**, stimulator of interferon genes (STING) **(B)**, and IFI16 **(C)** *in vitro* 72 hours after IR with 4, 8, or 3x8 Gy, normalized to housekeeping genes (GUSB and B2M) and respective controls (n = 3). **(D–F)** Fold change in expression of cGAS **(D)**, STING **(E)**, and IFI16 **(F)** in tumor cells *in vivo* 72 hours after IR with 8 or 3x8 Gy, normalized to housekeeping genes (GUSB and B2M) and respective controls (n = 5). **(G–I)** Fold change in expression of cG ing **(H)**, and p204 **(I)** in tumor cells *in vivo* 72 hours after IR with 8 or 3x8 Gy, normalized to housekeeping genes (BA and GAPDH) and respective controls (n = 5). Data is represented as mean ± standard error of the mean (SEM).

We investigated how different PSCC cell lines respond to IR in terms of cytosolic accumulation of dsDNA. Our findings showed that dsDNA accumulation was both time- and dose-dependent, with the highest number of dsDNA spots observed 72 hours after IR with a dose of 3x8 Gy in all tested cell lines. The UPCI:SCC090 cell line exhibited fewer dsDNA spots than the other cell lines (Figure 3A–B, Supplementary Figure S1). Based on this observation, we explored the effect of cytosolic dsDNA accumulation on the activation of DNA sensors. *ĩn vitro*, we found that the upregulation of cytosolic DNA sensors in response to IR is also dose- and time-dependent, following the pattern of accumulation of dsDNA in the cytosol of cells with the most significant alterations occurred 72 hours post-IR at a dose of 3x8 Gy (Figure 4, Supplementary Figure S2–S3). In UPCI:SCC090 cells, an upregulation was noted solely in the cGAS and STING following exposure to IR at 3x8 Gy. In the other PSCC cell lines, we observed a trend toward increased expression of cGAS, STING, and DDX60 after IR. More substantial fold changes were seen in DAI and RIG-I, which showed moderate upregulation across PSCC lines. Furthermore, FaDu and UM-SCC-6 cells lines differed in their activation of DAI and DDX60 (Figure 4A–C, Supplementary Figure S3). We extended our analysis to *in vivo* studies, examining the activation of cytosolic DNA sensors 72 hours after IR with doses of 8 or 3x8 Gy in both tumor cells and TME. Statistically significant differences were observed in the activation of cytosolic DNA sensors within tumor cells between the UPCI:SCC090 and HPSCC tumor models. In UPCI:SCC090 tumor cells, cGAS and STING were upregulated post-IR, consistent with *in vitro* data, and their levels were significantly higher than in HPSCC models (Figure 4D–E). Conversely, such activation of Sting and cGas was absent in the TME of UPCI:SCC090 (Figure 4G–H). In HPSCC tumor cells, overall upregulation of DNA sensors following IR was minimal, with no significant differences observed between the 2A3 and FaDu models (Figure 4D–F; Supplementary Figure S3). However, in the TME, we observed differential expression of p204, DAI, and DDX60 between FaDu and 2A3 tumors (Figure 4G–I; Supplementary Figure S3). Although the observed fold changes were relatively small, this may be partly due to high Ct values in the qPCR analysis, which indicate low baseline expression of these sensors in the tumor tissue.

# = indicates p < 0.05 for comparisons between UPCI:SCC090 and other cell lines or tumor models; * = indicates p < 0.05 for comparisons between IR doses within the same cell line or tumor model; o = indicates p < 0.05 for comparisons between FaDu and 2A3 models; + = indicates p < 0.05 for comparisons between UM-SCC-6 and FaDu

### Cytokine upregulation after irradiation is absent in HPV16-positive OPSCC tumors despite cGAS-STING activation

**FIGURE 5.**
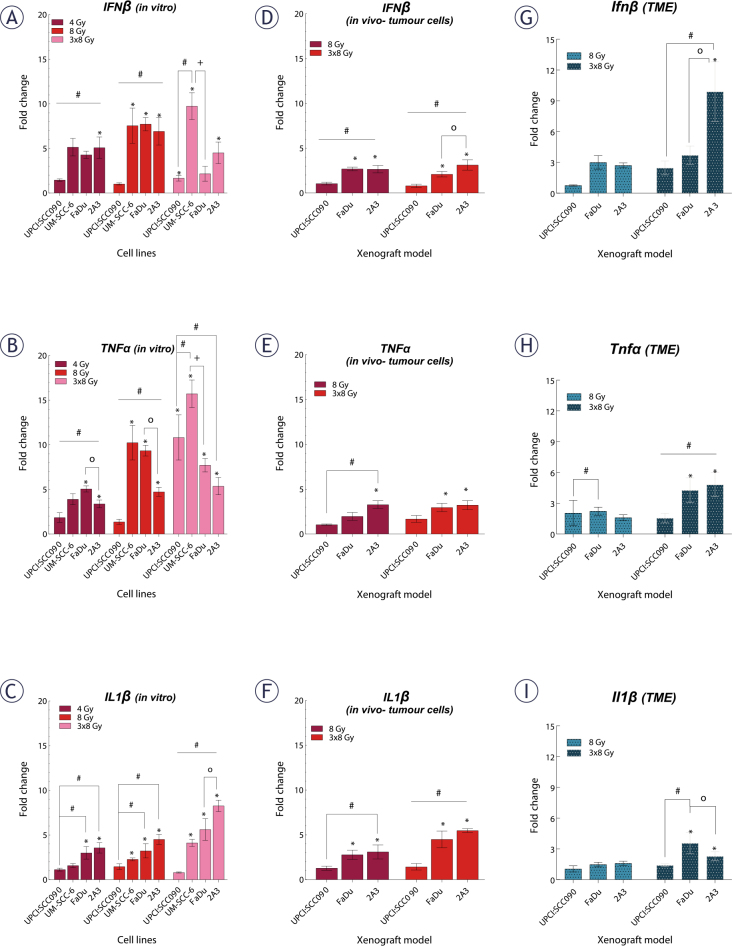
Effect of irradiation (IR) on cytokine gene expression in tumor cells and tumor microenvironment (TME) of pharyngeal squamous cell carcinoma (PSCC). **(A–C)** Fold change in expression of IFNβ **(A)**, tumor necrosis factor (TNF)α **(B)**, and IL1β **(C)** *in vitro* 72 hours after IR with 4, 8, or 3x8 Gy, normalized to housekeeping genes (GUSB and B2M) and respective controls (n = 3). **(D–F)** Fold change in expression of IFNβ **(D)**, TNFa **(E)**, and IL1 β **(F)** in tumor cells *in vivo* 72 hours after IR with 8 or 3×8 Gy, normalized to housekeeping genes (GUSB and B2M) and control (n = 5). **(G–I)** Fold change in expression of Ifnβ **(G)**, TNFα **(H)**, and Il1β **(I)** in the TME *in vivo* 72 hours after IR with 8 or 3–8 Gy, normalized to housekeeping genes (BA and GADPH) and control (n = 5). Data is represented as mean ± standard error of the mean (SEM). # = indicates p < 0.05 for comparisons between UPCI:SCC090 and other cell lines or tumor models; * = indicates p < 0.05 for comparisons between IR doses within the same cell line or tumor model; o = indicates p < 0.05 for comparisons between FaDu and 2A3 models; + = indicates p < 0.05 for comparisons between UM-SCC-6 and FaDu

Following the observation that IR induces dsDNA release in cytosol and activates DNA sensing pathways, we examined the expression of downstream cytokines. We found that cytokine upregulation after IR *in vitro* was dose- and time-dependent, just like the upregulation of cytosolic DNA sensors. Changes were predominantly observed 72 hours after a 3x8 Gy regimen (Figure 5A–C, Supplementary Figure S4). The UPCI:SCC090 cell line showed no upregulation in IL1β or IFNβ gene expression, regardless of the IR dose or time, compared to other cell lines. Conversely, a significant upregulation of the TNFα was observed 72 hours post-IR with 3x8 Gy in this cell line (Figure 5B).

Next, we investigated cytokine gene expression 72 hours after IR with 8 or 3x8 Gy in both tumor cells and the TME of PSCC tumors on mRNA level (Figure 5D–I). Our findings indicated that in UPCI:SCC090, there was no upregulation of any cytokines in tumor cells after IR with 8 or 3x8 Gy. However, HPSCC tumor models 2A3 and FaDu showed significant upregulation of all three cytokines in tumor cells. These models differed only in IFNβ response (Figure 5F). No upregulation of cytokine mRNA after IR was observed in the TME of the UPCI:SCC090 tumor model. Similar to tumor cells, statistically significant upregulation of all three cytokine mRNAs after IR was observed for 2A3 and FaDu in the TME (Figure 5G–I). Finally, we assessed cytokine production at the protein level by immunofluorescent staining of frozen tumor sections for IL1β, IFNβ, and TNFα. A statistically significant difference in IL1β levels was observed between UPCI:SCC090 and HPSCC tumors in control samples but not following IR with 3×8 Gy. HPSCC tumor models showed differences in TNFα levels across all experimental groups (Supplementary Figure S5).

### Innate immune infiltration occurs only in HPVI6-unrelated tumors after fractionated irradiation

**FIGURE 6.**
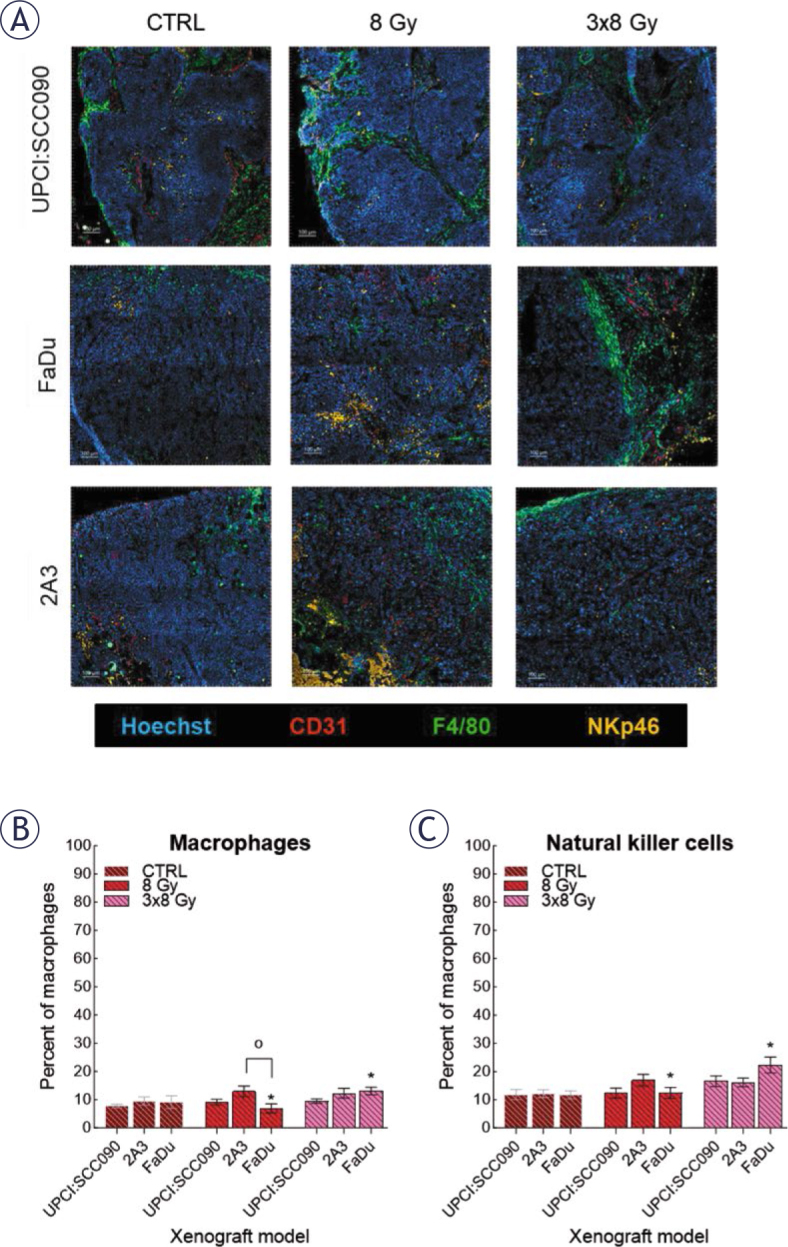
Response of the cellular innate immune system to irradiation (IR). **(A)** Frozen tumor sections were stained with anti-CD31 (red, Alexa 647), anti-F4/80 (green, Alexa 488), anti-natural killer cells (NK)p46 (orange, Cyanine 3), and Hoechst 33342 (blue). Scale bar: 100 μm. **(B)** Percentage of macrophages in tumor models before and after IR was determined by anti-F4/80 (calculated as the number of macrophages divided by the number of tumor cells). **(C)** Percentage NK cells in tumor models before and after IR was determined by anti-NKp46 (calculated as the number of NK divided by the number of tumor cells). Data are presented as mean ± standard error of the mean (SEM). # = indicates p < 0.05 for comparisons between UPCI:SCC090 and other cell lines or tumor models; * = indicates p < 0.05 for comparisons between IR doses within the same cell line or tumor model; o = indicates p < 0.05 for comparisons between FaDu and 2A3 models

Lastly, we investigated the cellular innate immune system’s response to IR in different tumor models. Frozen tumor sections collected 72 hours after IR with either 8 or 3x8 Gy were used for analysis. Samples were immunofluorescently stained for macrophages (F4/80 expression) and natural killer cells (NK; NKp46 expression) (Figure 6A). Our analysis demonstrated no statistically significant differences in macrophages and NK cell infiltration among the tumor models, regardless of whether they were control or IR-treated. However, we observed increased infiltration of both macrophages and NK cells in the FaDu model following the fractionated IR regime of 3x8 Gy (Figure 6B and C).

## Discussion

This study explored why HPV16-related OPSCC exhibits enhanced radiosensitivity, focusing on cytosolic DNA sensing pathways across HPV16-related and unrelated PSCC tumor models. Although we initially hypothesized that modulation of cytosolic DNA sensing pathways and innate immune responses by HPV16 oncoproteins E6 and E7 could explain the enhanced radiosensitivity of HPV16-related OPSCC, our findings suggest otherwise. We observed distinct baseline expressions of cytosolic DNA sensors and cytokines in HPV16-related OPSCC compared to other PSCCs, with HPV16-related OPSCC model exhibiting characteristics indicative of a suppressed STING pathway. After IR, expression of cytosolic DNA sensors and cytokines remained relatively unchanged in HPV16-related OPSCC, except for cGAS and STING sensors, whereas other PSCC models showed a time- and dose-dependent increase. The innate immune response to IR did not differ significantly across tumor models. Thus, our findings suggest cytosolic DNA sensing pathways and the innate immune response do not enhance radiosensitivity in HPV16-related OPSCC.

The response of cytosolic DNA sensing pathways to HPV16 infection is complex. We observed lower baseline STING expression in HPV16-related OPSCC tumor cells compared to other models, yet its activator, cGAS, showed no such difference. Previous studies demonstrated that HPV16 oncoproteins E6 and E7 suppress the cGAS-STING sensing pathway, aiding immune evasion.^18–20^ Despite this suppression, we observed higher baseline expression levels of cytokines IFNβ and TNFα in tumor cells, indicating pathway activation. Previous research demonstrated that even when IFN-inducing pathways, including cGAS-STING, are inhibited, TNFα can independently activate cGAS via mitochondrial DNA release.^21,22^ This explains why, despite the inhibition of E6 and E7 oncoproteins, the baseline expression of cGAS is not decreased in the HPV16-related OPSCC tumor model. TNFα and IFNβ are also released upon activation of IFI16, which was elevated in the HPV16-related OPSCC tumor model compared to other models. Similarly, IFI16, like cGAS-STING, detects viral DNA and triggers IFNβ induction and TNFα release from macrophages.^23,24^ Our data indicate TNFα expression is lower *in vitro*, but higher *in vivo*, as macrophages are present there. Interestingly, down regulation of IFI16 resulted in an increased release of IL1β, crucial for innate immune defenses and tumor radiation responses.^25,26^ Baseline IL1β expression was notably lower in both tumor cells and TME of HPV16-related OPSCC compared to other models, likely due to the activation of IFI16 by HPV16. Notable baseline differences in DNA sensors and cytokines between HPV16-related OPSCC and HPV16-related HPSCC, despite both containing HPV16 E6 and E7, are intriguing. This variance might stem from differences in the immune cell composition between oropharyngeal and hypopharyngeal tissues. However, the exact mechanisms underlying the tissue-specific response to HPV16 viral DNA in the pharynx are still unclear and require further investigation. Our data show differences in baseline expression of cytosolic DNA sensors and cytokines between HPV16-related OPSCC and other tumor models.

Cytosolic DNA sensing pathways play a key role in pathogen defense and responses to cellular damage. IR has been shown to activate these pathways by promoting the release of DNA into the cytosol. Vanpouille-Box *et al*. demonstrated that fractionated doses of 3×8 Gy optimally activate these pathways, while a single high dose (20 Gy) induces TREX1-mediated degradation of cytosolic dsDNA, suppressing immunogenic signaling.^27^ In our previous study, we found that 8 Gy induced the highest upregulation of DNA sensors *in vitro*.^28^ Based on these findings, we selected 8 Gy and 3×8 Gy to investigate DNA sensor activation in PSCC models. Our results indicated IR-induced upregulation of cytosolic DNA sensors and cytokines was time- and dose-dependent. In HPV16-related OPSCC, upregulation of sensors cGAS and STING occurred only 72 hours after IR with 3x8 Gy, whereas expression of other DNA sensors remained unchanged. As previously mentioned, the STING sensor was initially suppressed by HPV16 E6 and E7 oncoproteins.^19,29^ As demonstrated, this inhibition was later disrupted by fractionated IR doses. Previous studies showed IR causes an increased expression of HPV16 E6 and E7 oncoproteins, which in our case would mean that the STING sensor would continue to be suppressed, which is not the case.^30–33^ We hypothesize fractionated IR disrupts HPV16 DNA rapid repair, resulting in suppressed expression of E6 and E7, leading to STING sensor activation. Despite activating the cGAS-STING pathway, cytokines IFNβ and TNFα mRNA levels remained unchanged *in vivo*, possibly due to the inactive IFI16 sensor. Expression of the cytokine IL1β remained low in HPVI6-related OPSCC tumor cells. Previous studies have linked overexpression of IL1β to radioresistance. In HPV16-related OPSCC IL1β was downregulated, which could be one reason for better radiosensitivity of mentioned model.^26,34–36^ In HPV16-related OPSCC model, we did not observe a significant upregulation of DNA sensors or cytokines within the TME, suggesting that the enhanced radiosensitivity of this model is not mediated by TME-related factors. Conversely, other tumor models exhibited increased DNA sensor and cytokine expression in tumor cells and TME, irrespective of HPV16 status.

A notable difference between HPV16-related OPSCC and other tumor models was the significantly lower cytosolic dsDNA release after IR in HPV16-related OPSCC model. This might result from upregulation of the three prime repair exonuclease (TREX1). Elevated TREX1 expression has been observed in HPV-associated cervical cancer, facilitating tumor proliferation and progression by impeding p53 functionality.^37^ Furthermore, TREX1 also acts as a safeguard mechanism; under high IR doses, it is activated to degrade cytosolic DNA and thereby preventing activation of cytosolic DNA sensing pathway and subsequent immune response.^28,38^ We hypothesize that activation of cytosolic DNA sensors in HPV16-related OPSCC is influenced by both HPV16 oncoproteins E6 and E7 as well as TREX1. While the former pair initially suppress immune recognition, TREX1, whose expression might also be elevated in HPV16-related OPSCC, can be triggered even at lower IR doses. These activation dynamics may contribute to the muted response of cytosolic sensors in this tumor model, consequently resulting in the absence of cytokine release.

Activation of cytosolic DNA sensors usually induces cytokine release, stimulating immune responses to IR.^39–41^ Previous studies reported differences in macrophage and NK cell levels between HPV16-related OPSCC and HPV16-unrelated PSCC, which we did not observe in our mouse xenograft model.^42,43^ Following 3x8 Gy IR, increased infiltration of both macrophages and NK cells occurred only in the HPV16-unrelated HPSCC model. Activation of the innate immune system partially occurred only in this model, where we had also observed activation of both cytosolic DNA sensors and cytokines. In contrast, no similar effect occurred in HPV16-related HPSCC tumor model, despite evident activation of cytosolic DNA sensing molecular pathways. This may be due to HPV16 oncoproteins E6 and E7, which still suppress immune system but not in the same way as in HPV16-related OPSCC. Previous studies have shown better IR response in HPV16-related OPSCC than HPV16-unnrelated PSCC, which partially aligned with our findings.^44,45^ *In vitro*, the HPV16-related OPSCC cell line was the most radiosensitive, while no significant differences were observed among other PSCC cell lines. The HPV16-related OPSCC tumor group also showed improved survival following a single 8 Gy IR dose compared to the HPSCC group subjected to the same IR regimen. However, no survival differences were noted among groups that received a fractionated 3x8 Gy dose, possibly due to excessive overall dose toxicity inducing tumor cures. Our findings suggest that the absence of activation of cytosolic DNA sensing pathways in HPV16-related OPSCC leads to diminished innate immunity and therefore does not play a role in its enhanced radiosensitivity.

Despite an attempt to illuminate the role of cytosolic DNA sensing pathways in response to IR in PSCC tumor models, as comprehensively as possible, we must address potential limitations of the present research. One limitation is the unsuccessful engraftment of the UM-SCC-6 tumor line, despite multiple attempts. Second, although the 2A3 cell line is HPV16-related, it lacks the complete viral genome and may not fully represent HPV16-associated biology. It was developed by transfecting FaDu cells with HPV16 E6 and E7 oncogenes via the PA317 LXSN 16E6/E7 vector and remains the only available human HPV16-related HPSCC cell line. Another limitation is the use of a single HPV16-related OPSCC cell line.^31^ Inclusion of additional models would have strengthened the study and improved our understanding of the heterogeneity among HPV16-positive tumors. Unfortunately, very few HPV16-related cell lines of oropharyngeal origin are commercially available. Next, knockdown of cGAS and STING in tumor models would have been useful to directly assess their functional impact. Furthermore, quantification of cytokines using western blotting and immune cell populations with flow cytometry would provide further support to delineate the effects of DNA sensing pathways in context of immune system activation. However, since the activation of DNA sensors did not lead to cytokine induction or immune response in our models, we decided not to pursue this approach in the current study. Finally, the adaptive immune system plays a significant role in the response to IR. However, immunocompromised mice that we used cannot activate it due to the absence of a thymus, which can lead to reduced radiosensitivity of specific tumor models. On the other hand, this way we were able to investigate how the innate immune system itself contributes to sensitivity to IR.

The key finding of our research was that the involvement of cytosolic DNA sensing pathways and innate immune system do not increase radiosensitivity of HPV16-related OPSCC. In PSCC models, DNA sensors and cytokine expression varied depending on IR dose and fractionation, with the most notable changes observed 72 hours after fractionated 3x8 Gy. The HPV16-related OPSCC tumor model showed upregulation of cGAS and STING, without corresponding cytokine induction, suggesting potential for future studies using STING agonists or antagonists to modulate tumor response. In addition, we detected differences in cytosolic accumulation of dsDNA across cell lines, which may be influenced by TREX1 activity. Furthermore, our results partially refute the notion that the activation of cytosolic DNA sensing pathways depends on HPV16 status, as similar activation patterns were observed in both HPV16-related and unrelated HPSCC tumor models. Additional research exploring the interplay between adaptive immunity and cytosolic DNA sensing pathways could help clarify the mechanisms underlying the enhanced radiotherapy responses observed in patients with HPV16-related OPSCC. The recently developed HPV16-positive murine model MOC-1 could be particularly valuable in this context, as it enables the investigation of adaptive immune responses to IR.^46^

## Supplementary Material

Supplementary Material Details
