# Supplementary material for: Human papillomavirus-related oropharyngeal squamous cell carcinoma exhibits enhanced radiosensitivity despite limited activation of cytosolic DNA sensing pathways and innate immune responses
Source: Radiol Oncol. 2025 Dec 16;59(4):566–78. doi: 10.2478/raon-2025-0057 (PMC12707452; doi:10.2478/raon-2025-0057)
Supplement: Supplementary file 1 — Supplementary Material Details [file raon-2025-0057_sm.pdf]

Human papillomavirus-related oropharyngeal squamous cell carcinoma exhibits enhanced radiosensitivity despite limited activation of cytosolic DNA sensing pathways and innate immune responses

Kristina Levpuscek, Tanja Jesenko, Tilen Komel, Simona Kranjc Brezar,
Gregor Sersa, Maja Cemazar, Primoz Strojan

doi: 10.2478/raon-2025-0057

Supplementary materials and methods

Real-time quantitative polymerase chain reaction (RT-qPCR)

DNA sensors and cytokine mRNA expression *in vitro* and *in vivo* were detected by RT-qPCR. For an *in vitro study*, cells were seeded in T25 flasks (Corning, New York, USA). After attachment, cells were irradiated with 4, 8, or 3×8 Gy, except for the control group. According to the manufacturer's instructions, the treated cells were harvested 48 or 72 hours after treatment. Total RNA was isolated from cell pellet using a peqGOLD Total RNAKit (VWR, West Chester, PA, USA). In the case of tumors, TRIzol Reagent (Thermo Fischer Scientific) was used for homogenization and extraction, followed by isolation of RNA with peqGOLD Total RNAKit from aqueous portion of the sample. The concentration and quality of RNA were determined by absorbance using a microplate reader (Cytation 1, BioTek Instruments, Winooski, VT, USA). SuperScript VILO cDNA Synthesis Kit (Invitrogen, Thermo Fisher Scientific) was used for reverse transcription of 1000 ng of RNA. Afterward, cDNA was diluted ten-fold and stored at -20 °C until further analysis. RT-qPCR was performed with QuantStudio 3 Real-Time PCR System (Thermo Fisher Scientific). The 20 µl reaction mixture consisting of 10 µl of SYBR Green Master Mix (Thermo Fisher Scientific), 4.6 µl of nuclease-free water, 0.4 µl of appropriate custom or commercial primer pairs (Integrated DNA Technologies, Coralville, IA, USA) (Table S1), and 5 µl of cDNA was amplified according to the following protocol: 2 minutes at 50 °C, 10 minutes at 95 °C, 40 cycles of 15 seconds at 95 °C, 1 minute at 60 °C. Afterward, data were analyzed using QuantStudio's software. Two human and two mouse housekeeping genes were used for normalization. To distinguish human tumor cells from TME in tumor samples, mouse primer pairs were used (Supplementary Table S1). The threshold cycle number (Ct) was measured automatically. Each sample was performed in two technical parallels. Relative expression was calculated using

the ΔCt = Ct (gene) - Ct (average of housekeeping genes). Fold change in gene expression was calculated using the 2-ΔΔCt method where ΔΔCt = ΔCt (gene of the treated group) - ΔCt (gene of the control group). The nondetermined (N.D.) value was annotated above the 40th cycle.

SUPPLEMENTARY TABLE S1. List of mouse and human real-time quantitative polymerase chain reaction (RT-qPCR) primers

Primer	Target	Sequence (5′-3′)
mouse	Ba_F	GAAGTGTGACGTTGACATCC
mouse	Ba_R	ACTCATCGTACTCCTGCTTG
mouse	Gapdh_F	TTCACCACCATGGAGAAGGC
mouse	Gapdh_R	GGCATGGACTGTGGTCATGA
mouse	cGAS_F	GTGAGGACCAATCTAAGACGAG
mouse	cGAS_R	AGCATGTTTTCTCTATCCCGTG
mouse	Dai_F	TGCTTTCTAGAGGACGCCACCATT
mouse	Dai_R	TGGCTTCAGAGCTTGTACCTGTGT
mouse	Sting_F	GTCCTCTATAAGTCCCTAAGCA
mouse	Sting_R	AAGATCAACCGCAAGTACCC
mouse	Ddx60_F	ACTGGAACACTCGCTTTGG
mouse	Ddx60_R	GAAGTAGACATCACCCAACAGG
mouse	p204_F	CCAGTCACCAATACTCCACAG
mouse	p204_R	GAGCACCATCACTGTCAGG
mouse	Rig-I_F	GAGCCAGCGGAGATAACAATA
mouse	Rig-I_R	CCCACGTACTCATAGAGAATGAC
mouse	Ifnβ1_F	TGGCCATCCAAGAGATGCTCCAGA
mouse	Ifnβ1_R	AGAAACACTGTCTGCTGGTGGAGT
mouse	Il1β_F	AGTTGACGGACCCCAAAAGA
mouse	Il1β_R	TGCTGCTGCGAGATTTGAAG
mouse	Tnfa_F	CCCTCCAGAAAAGACACCATG
mouse	Tnfa_R	GTCTGGGCCATAGAACTGATG
human	hB2M_F	GGCATTCCTGAAGCTGACAG
human	hB2M_R	TGGATGACGTGAGTAAACCTG

human	hGUSB_F	AGGTGATGGAAGAAGTGGTG
human	hGUSB_R	AGGATTTGGTGTGAGCGATC
human	hRIG-I_F	TGGCATATTGACTGGACGTG
human	hRIG-I_R	CACTGGCTTTGAATGCATCC
human	hcGAS_F	AGGAAGCAACTACGACTAAAGC
human	hcGAS_R	TCACAGCACGTTTTAGATTTTCC
human	hIFI16_F	TGCCAGCGTAACTCCTAAAATC
human	hIFI16_R	CCACTTCCATCTTCCCTGTATT
human	hDAI_F	CAGCTACAATTCCAGAGACCC
human	hDAI_R	TCTTTTGCTGTCCTCATTCCC
human	hSTING_F	CTTCACTTGGATGCTTGCC
human	hSTING_R	CCGATGTAATATGACCATGCCAG
human	hDDX60_F	TTATTCCCGACACATGGCAG
human	hDDX60_R	CTCTCCTTCAGCACTTTCTCC
human	hTNFa_F	ACTTTGGAGTGATCGGCC
human	hTNFa_R	GCTTGAGGGTTTGCTACAAC
human	hIFNb_F	CTAACTGCAACCTTTCGAAGC
human	hIFNb_R	GGAAAGAGCTGTAGTGGAGAAG
human	hIL-1β_F	CCATGGCAGAAGTACCTGAG
human	hIL-1β_R	CCTGGAAGGAGCACTTCATC

Accumulation of double-stranded DNA (dsDNA) in the cytosol of cells

Accumulation of dsDNA in the cytosol of cells was identified by immunostaining. A specific number of cells (5000 cells/well for UM-SCC-6, FaDu and 2A3 or 10000 cells/well for UPCI:SCC090) were seeded in 12-well chambers (Ibidi, Gräfelfing, Germany) and allowed to adhere overnight. Plating density was adapted to the doubling times of cell lines. The experimental groups were irradiated with 4, 8, or 3x8 Gy except for the control group. At 48 or 72 hours after irradiation (IR), cells were rinsed with Hanks' Balanced Salt Solution (HBSS, calcium, magnesium, Gibco, Thermo Fisher Scientific) for 5 minutes at room temperature (RT) and then fixed with 4% paraformaldehyde (PFA; Alfa Aesar, Thermo Fisher Scientific) for 15 minutes at 37°C. After three washing steps in HBSS, cells' plasma membranes were stained with 1 µg/mL Wheat Germ Aglutinin (Alexa Fluor 647, Thermo Fisher Scientific) for 10 minutes at RT. Cells were rinsed twice with HBSS for 5 minutes at RT and then permeabilized with nonionic detergent 0.01% or 0.1% Tween 20 (Sigma-Aldrich, Missouri, MO, USA) for 10 minutes at RT. 0.1% Tween 20 was used for UM-SCC-6 and UPCI:SCC090, on the other hand 0.01% Tween 20 was used for FaDu and 2A3. The concentrations of Tween 20 permeabilized the plasma membrane, but were not high enough to permeabilize the nuclear membrane in order to reduce the signal coming from the nucleus that would impair the identification of dsDNA in the cytosol. After being rinsed twice with phosphate-buffered saline (PBS) for 5 minutes at RT, nonspecific binding was blocked with blocking buffer (0.01% Tween 20, 5% donkey serum, 22.52 mg/mL glycine in PBS) for 1 hour at RT. Cells were incubated with primary anti-dsDNA antibody (35I9 DNA; 1:1000; Abcam, Cambridge, UK) diluted in blocking buffer overnight at 4°C. The following day, cells were rinsed three times with PBS for 5 minutes at RT. The incubation with secondary antibody donkey anti-mouse IgG H&L (Alexa Fluor 488; 1:500; Abcam) in PBS lasted 1 hour at RT. After thorough washing, cell nuclei were stained with 3µg/mL of Hoechst solution (Hoechst 33342, Thermo Fisher Scientific) in PBS for 10 minutes at RT. Afterward, cells were rinsed three times with PBS for 5 minutes at RT. The coverslips were mounted on slides using ProLong Glass Antifade Mountant (Thermo Fisher Scientific). Immunofluorescence microscopy was performed using LSM 800 confocal microscope (Carl Zeiss, Oberkochen, Germany) with a 63×oil immersion objective. Images were further analyzed using the Imaris software (Bitplane, Zurich, Switzerland). The number of dsDNA spots per cell was quantified by dividing the number of spots with the number of cell nuclei on the image.

SUPPLEMENTARY TABLE S2. List of antibodies used for immunostaining dsDNA in the cytosol of cells

Target	Supplier	Cat. No.	Dilution
dsDNA	Abcam	ab27156	1:1000
Donkey Anti-Mouse IgG H&L Antibody (Alexa Fluor® 488)	Abcam	ab150105	1:500

Immunofluorescence staining of tumors

Fourteen-micrometer-thick frozen sections were cut using a Leica CM1850 cryostat (Leica Biosystems, Wetzlar, Germany) and mounted onto the Superfrost plus glass slides (ThermoFisher Scientific). Slides were then stained with primary and secondary antibodies for immune cells and cytokines (Table S3). The staining protocol for immune cells and cytokines began with drying the slides for 10 minutes at 37°C, followed by a 5-minute wash in 1xPBS. Afterward, sections of slides were blocked and permeabilized in a Blocking buffer 1 (5% Tween 20, 5% donkey serum, 22.52 mg/mL glycine in PBS) for 1 hour at RT in a humidified chamber, followed by two 5-minute wash in Blocking buffer 2 (2% donkey serum, 22.52 mg/mL glycine in PBS). Slides stained for immune cells and cytokines were then incubated with primary antibodies overnight at 4°C in a Blocking buffer 2 within a humidified chamber (Table S3). Following three 10-minute 1xPBS washes the next day, slides were incubated with secondary antibodies for 1 hour at RT in a humidified chamber and then washed three times in 1xPBS (Table S3). Nuclei were stained with Hoechst 33342 solution (3 μg/mL in 1xPBS) for 5 minutes in the dark, followed by 45-minute 1xPBS wash. Slides were then mounted with ProLong™ Glass Antifade Mountant (Thermo Fisher Scientific). Imaging was performed on three tumor samples per group using an LSM 800 confocal microscope (Carl Zeiss) with a 10x or 20x objective, utilizing Hoechst 33342, Alexa Fluor 488, Cyanine 3, and Alexa Fluor 647 fluorophores, excited at wavelengths of 405 nm, 488 nm, 561 nm, and 640 nm, respectively. Emission was captured using a Gallium Arsenide Phosphide (GaAsP) detector, combined with variable dichroic and channel-specific filters: 410–545 nm (Hoechst 33342), 488–545 nm (Alexa Fluor 488), 565–620 nm (Cyanine3), and 645–700 nm (Alexa Fluor 647). The images were visualized and analyzed with Imaris software and CellProfiler.

SUPPLEMENTARY TABLE S3. List of antibodies used for *in vivo* experiments

Primary antibody	Dilution	Secondary antibody	Dilution
Anti-CD31 antibody (Abcam; ab28364)	1:100	Donkey anti-Rabbit IgG (H+L) Highly Cross-Adsorbed Secondary Antibody, Alexa Fluor™ 647, Invitrogen™ (Fisher Scientific; A31573)	1:200
NKp46 goat a-mouse (Thermo Fischer Scientific; PA5-46986)	1:200	Cy™3 AffiniPure™ Donkey Anti-Goat IgG (H+L) (Jackson Immunoresearch; 705-165-147)	1:200
Rat anti-F4/80 antibody, clone BM8 (Thermo Fischer Scientific; 14-4801-82)	1:200	Donkey anti-rat IgG (Alexa Fluor® 488) (Jackson Immunoresearch; 712-545-150)	1:200
CD11b Monoclonal Antibody (M1/70), PE-Cyanine7, eBioscience™ (Thermo Fischer Scientific; 25-0112-82)	1:150	Cy™3 AffiniPure™ Donkey Anti-Goat IgG (H+L) (Jackson Immunoresearch; 705-165-147)	1:200
IFN beta Polyclonal Antibody (Thermo Fischer Scientific; PA5 20390)	1:150	Donkey anti-Rabbit IgG (H+L) Highly Cross-Adsorbed Secondary Antibody, Alexa Fluor™ 647, Invitrogen (Fisher Scientific; A31573)	1:200
Anti-IL1 beta antibody (Abcam; 9722)	1:200	Donkey anti-Rabbit IgG (H+L) Highly Cross-Adsorbed Secondary Antibody, Alexa Fluor™ 647, Invitrogen™ (Fisher Scientific; A31573)	1:200
Anti-TNF alpha antibody (Abcam; 6671)	1:200	Donkey anti-Rabbit IgG (H+L) Highly Cross-Adsorbed Secondary Antibody, Alexa Fluor™ 647, Invitrogen™ (Fisher Scientific; A31573)	1:200

Supplementary results

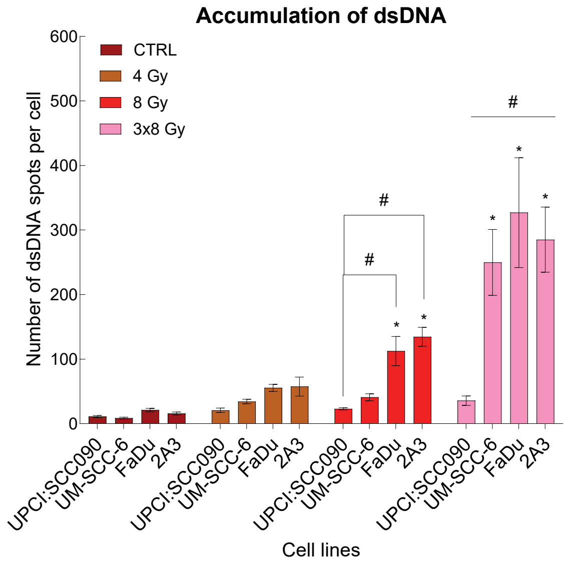

SUPPLEMENTARY FIGURE S1. Irradiation (IR)-induced accumulation of dsDNA in the cytosol of cells. Number of dsDNA spots in cytosol per cell 48 hours after IR with 4, 8, or 3x8 Gy (n = 8). Data is represented as mean ± standard error of the mean (SEM).

#= indicates $p < 0.05$ when comparing UPCI:SCC090 with the remaining cell lines; o = indicates $p < 0.05$ when comparing FaDu and 2A3 cell lines; + = indicates $p < 0.05$ when comparing UM-SCC-6 and FaDu cell lines; * = indicates $p < 0.05$ when comparing different IR doses (Gy) in the same cell line

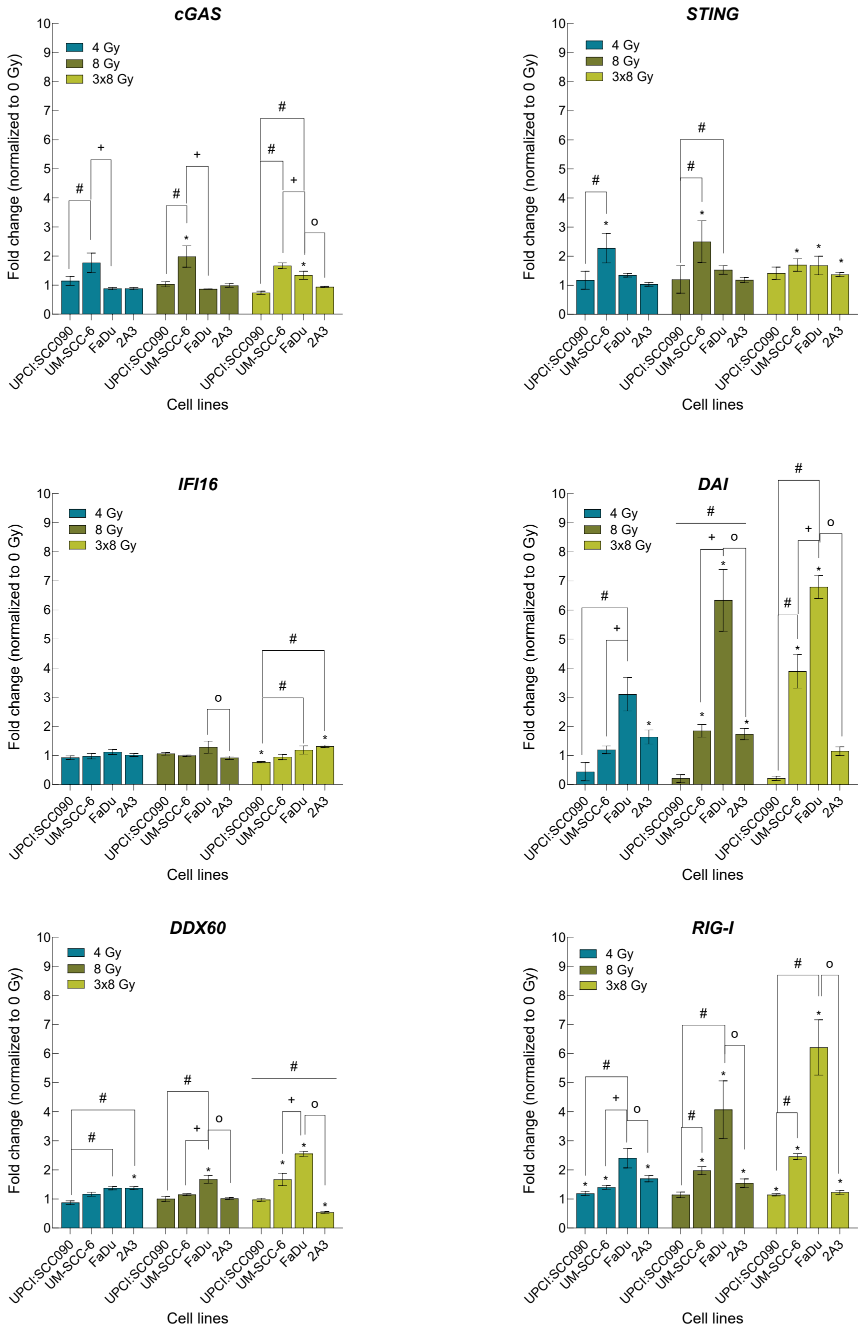

SUPPLEMENTARY FIGURE S2. Effect of irradiation (IR) on gene expression of cytosolic DNA sensors in tumor cells in vitro. Expression fold change of cytosolic DNA sensors in cells in vitro 48 hours after IR with 4, 8, or 3x8 Gy, normalized to housekeeping genes (GUSB and B2M) and control (n = 3). Data is represented as mean ± standard error of the mean (SEM).

# = indicates $p < 0.05$ when comparing UPCI:SCC090 with the remaining cell lines; o indicates $p < 0.05$ when comparing FaDu and 2A3 cell lines; + = indicates $p < 0.05$ when comparing UM-SCC-6 and FaDu cell lines; * = indicates $p < 0.05$ when comparing different IR doses (Gy) in the same cell line

cGAS = GMP-AMP synthase; STING = stimulator of interferon genes

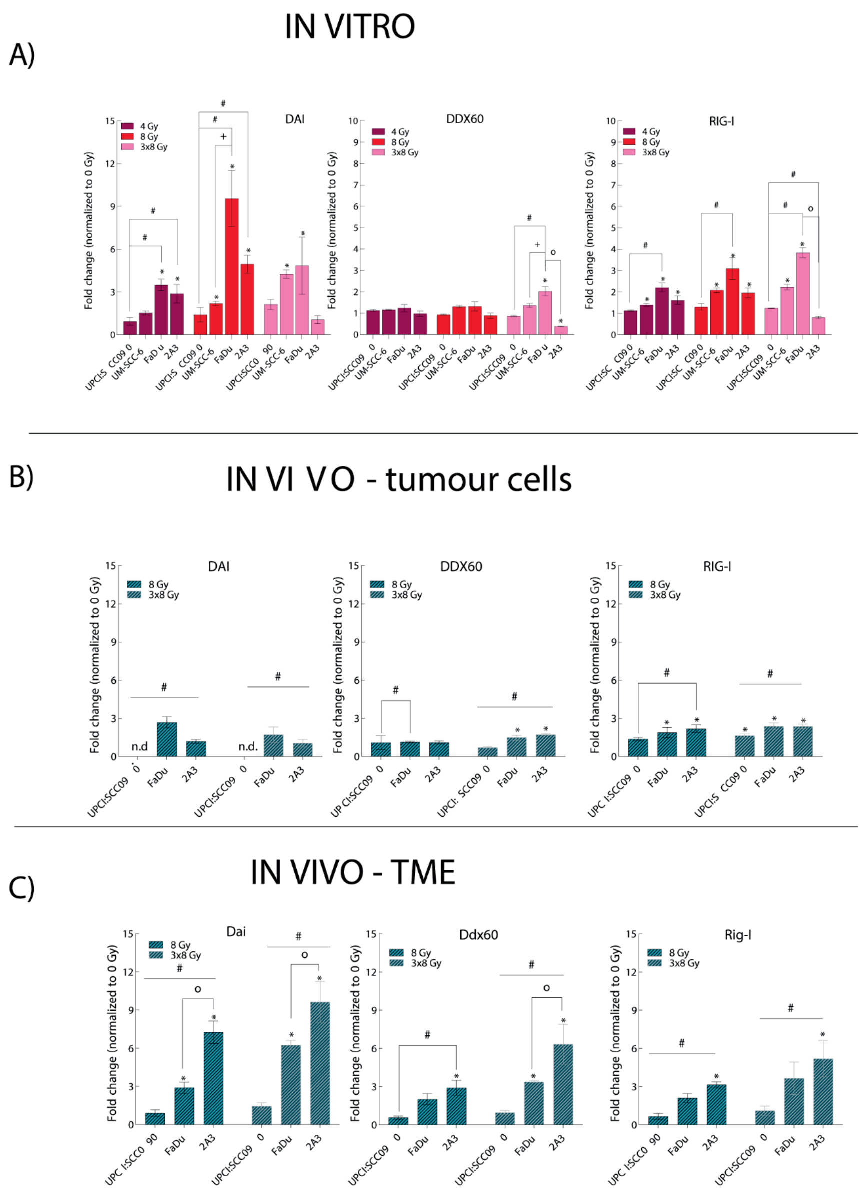

SUPPLEMENTARY FIGURE S3. Effect of irradiation (IR) on gene expression of cytosolic DNA sensors in tumor cells and tumor microenvironment of pharyngeal squamous cell carcinoma (PSCC). A) Expression fold change of cytosolic DNA sensors in cells in vitro 72 hours after IR with 4, 8, or 3x8 Gy, normalized to housekeeping genes (GUSB and B2M) and control. n = 3. B) Expression fold change of cytosolic DNA sensors in tumor cells in vivo 72 hours after IR with 8 or 3x8 Gy, normalized to housekeeping genes (GUSB and B2M) and control. n = 5. C) Expression fold change of cytosolic DNA sensors in TME in vivo 72 hours after IR with 8 or 3x8 Gy, normalized to housekeeping genes (BA and GADPH) and control. n = 5. Data are presented as mean ± SEM. Statistical significance $p < 0.05$ was determined by One-way ANOVA or Kruskal-Wallis test.

# = indicates $p < 0.05$ when comparing UPCI:SCC090 with the remaining cell lines/tumor models; o = indicates $p < 0.05$ when comparing FaDu and 2A3 cell lines/tumor models; + = indicates $p < 0.05$ when comparing UM-SCC-6 and FaDu cell lines; * = indicates $p < 0.05$ when comparing different IR doses (Gy) in the same cell line/tumor model

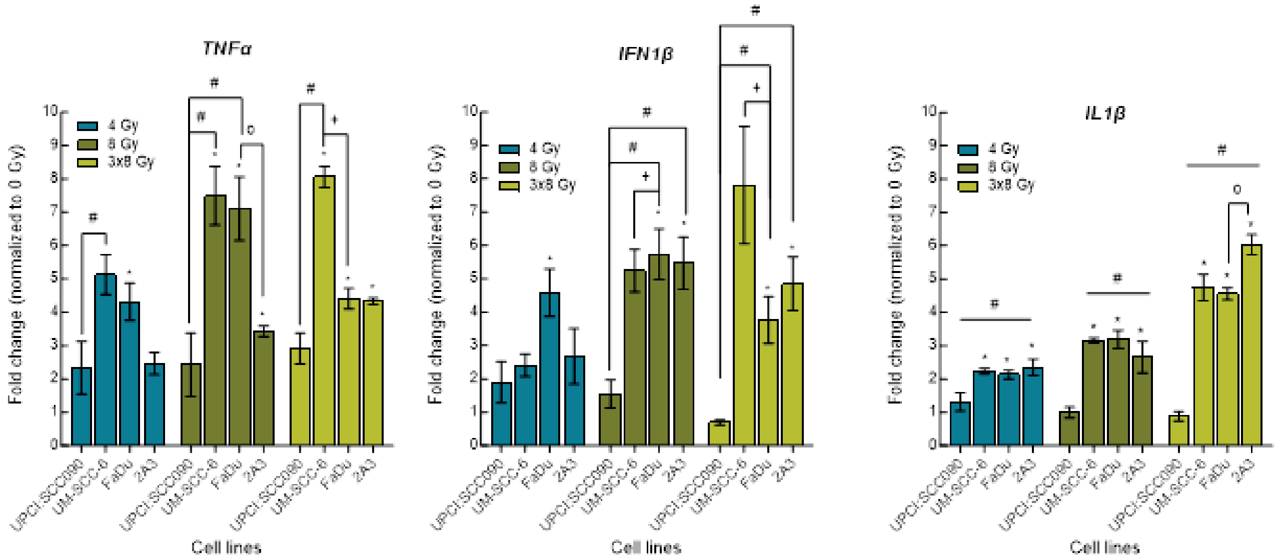

SUPPLEMENTARY FIGURE S4. Effect of irradiation (IR) on gene expression of cytokines in tumor cells in vitro. Expression fold change of cytokines in cells in vitro 48 hours after IR with 4, 8, or 3x8 Gy, normalized to housekeeping genes (GUSB and B2M) and control. n = 3. The gene expression of DNA sensing pathways was determined by RT-qPCR. The data is represented as mean ± SEM. Statistical significance $p < 0.05$ was determined by One-way ANOVA or Kruskal-Wallis test.

# = indicates $p < 0.05$ when comparing UPCI:SCC090 with the remaining cell lines; o = indicates $p < 0.05$ when comparing FaDu and 2A3 cell lines; + = indicates $p < 0.05$ when comparing UM-SCC-6 and FaDu cell lines; * = indicates $p < 0.05$ when comparing different IR doses (Gy) in the same cell line

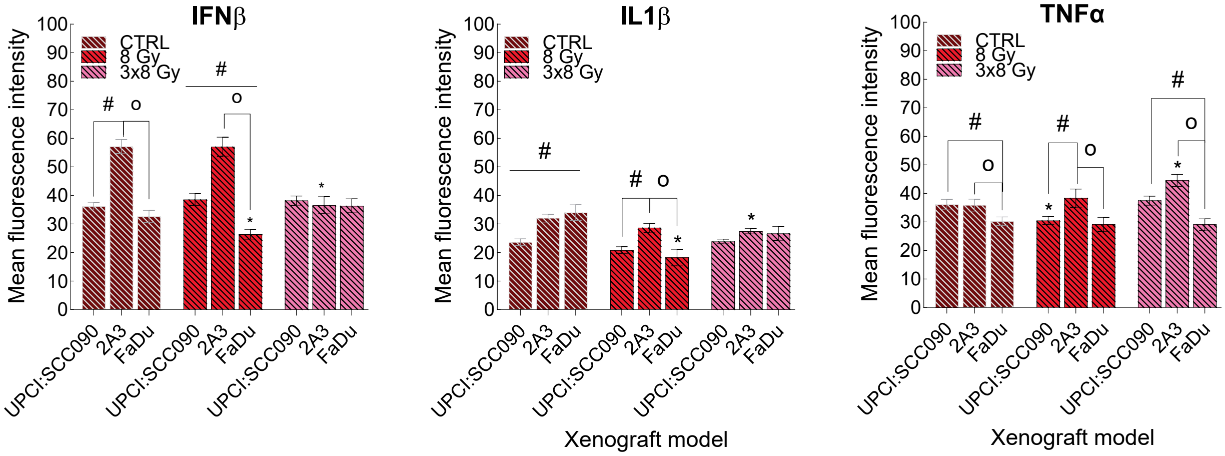

SUPPLEMENTARY FIGURE S5. Effect of irradiation (IR) on protein expression of cytokines in pharyngeal squamous cell carcinomas (PSCCs). Mean fluorescence intensity of cytokines in tumor sections 72 hours after IR with 8 or 3x8 Gy. Data is represented as mean ± SEM.

# indicates $p < 0.05$ when comparing UPCI:SCC090 with the remaining cell lines/tumor models; o = indicates $p < 0.05$ when comparing FaDu and 2A3 cell lines/tumor models; * = indicates $p < 0.05$ when comparing different irradiation doses (Gy) in the same cell line/tumor model
